# Prevalence of colonoscopy-related adverse events in older adults aged over 65 years: a systematic review and meta-analysis

**DOI:** 10.1097/JS9.0000000000002282

**Published:** 2025-01-29

**Authors:** Qing Lu, Xiu-He Lv, Li Tang, Hai-Lin Yan, Bi-Han Xia, Zhu Wang, Jin-Lin Yang

**Affiliations:** a Department of Gastroenterology and Hepatology, West China Hospital of Sichuan University, Chengdu, Sichuan, China; b Department of Gastroenterology and Hepatology, Sichuan University-Oxford University Huaxi Gastrointestinal Cancer Centre, West China Hospital of Sichuan University, Chengdu, Sichuan, China; c Department of Gastroenterology and Hepatology, Section of Nursing, West China Hospital of Sichuan University, Chengdu, Sichuan, China(Prof. Yang)

**Keywords:** adverse event, aged, colonoscopy, meta-analysis, octogenarians

## Abstract

**Background:**

This study aims to assess the occurrence of colonoscopy-related adverse events (AEs) in adults aged over 65 years, as there has been a significant increase in the prevalence of colonoscopies among the elderly compared to two decades ago.

**Methods:**

A comprehensive search was conducted on 3 June 2024, using the PubMed, Embase, and Cochrane Library databases. Meta-analyses were performed using the generalized linear-mixed model, and the results were presented as pooled rates with relevant 95% confidence intervals (CIs).

**Results:**

We retrieved a total of 15 417 records and included 13 population-based studies. The overall rates of colonoscopy-related perforation and bleeding in the elderly population were 7.8 (95% CI 5.5–11.2; *I*^2^ = 94%) and 23.5 (95% CI 9.0–61.3; *I*^2^ = 100%) per 10 000 colonoscopies, respectively. The “ > 80 years” group had a significantly higher risk of perforation (RR 2.55; 95% CI 1.15–5.66; *I*^2^ = 79%) and bleeding (RR 1.23; 95% CI 1.02–1.48; *I*^2^ = 0%) compared to the “65–80 years” group. For screening colonoscopies, the rates of perforation and bleeding were 8.5 (95% CI 7.1–10.2; *I*^2^ = 0%) and 27 (95% CI 9.0–81.0; *I*^2^ = 99%) per 10 000 colonoscopies, respectively. For diagnostic colonoscopies, the rates of perforation and bleeding were 18 (95% CI 16.2–20.0; *I*^2^ = 1%) and 16 (95% CI 8.1–31.3; *I*^2^ = 98%) per 10 000 colonoscopies, respectively. Compared to non-therapeutic colonoscopies, therapeutic procedures exhibited higher rates of both perforation (1.5 vs. 0.4 per 10 000 colonoscopies) and bleeding (7.1 vs. 0.5 per 10 000 colonoscopies). The prevalence of cardiopulmonary AEs in the elderly population is relatively high, although the definition used varies across different studies.

**Conclusions:**

We conducted a comprehensive analysis on the prevalence of AEs related to colonoscopy in older adults. Overall, the AE rates remain low. However, we emphasize the importance of enhancing safety protocols to further minimize risks, ensuring that the benefits of colonoscopy continue to outweigh the risks, especially for patients over the age of 80.

## Introduction

The phenomenon of global population aging is increasingly evident, characterized by a steady rise in the elderly demographic across nations worldwide. According to the World Population Prospects 2019 report, it is projected that by 2050, approximately one out of every six individuals globally will be aged 65 or above, representing an increase from the current ratio of one out of every eleven individuals in 2019[1]. Concurrently with this growth in the elderly population, there has been a notable rise in the prevalence of colorectal diseases. Notably, among individuals aged 65 to 75 years, the global incidence of colorectal cancer reached its peak in 2019[2]. Colonoscopy is widely recognized as the most effective technique for identifying and monitoring colorectal diseases. In particular, screening colonoscopy has shown significant associations with reduced risk and mortality related to colorectal cancer[3]. Consequently, there has been a significant surge in demand for colonoscopies compared to two decades ago.

**Highlights**

- The prevalence of colonoscopy-related perforation and bleeding in older adults aged over 65 years were 0.078% and 0.235%, respectively. The very elderly group (aged over 80 years) had a significantly higher risk of perforation and bleeding compared to the young-elderly group (aged 65 to 80 years).
- The prevalence of cardiopulmonary AEs related to colonoscopy was relatively high, albeit with varying definitions across studies.
- The findings of the present study emphasize the importance of rigorous precolonoscopy assessment, meticulous intra-procedural monitoring, and continuous post-colonoscopy surveillance in the elderly population.

Endoscopists have long been concerned about the potential increased risk of adverse effects (AEs) associated with advanced age during colonoscopy[4]. Given the limited physiological reserves and prolonged recovery in elderly individuals, additional post-procedural care may be necessary[5]. Regardless of the indication for colonoscopy, a thorough assessment of risks and benefits is essential when evaluating its necessity as patients age. Several studies consistently demonstrate an age-dependent increase in colonoscopy-related AEs; however, contrasting perspectives also argue for the safety of colonoscopy in elderly individuals^[6–9]^.

The existing systematic reviews of colonoscopy-related AEs fail to specifically address this issue in the elderly population^[4,10]^; furthermore, several guidelines lack quality control indicators pertaining to the safety of colonoscopy in older adults^[11,12]^. The limited number and small sample sizes of available studies posed challenges during our exploration of this topic. Therefore, this study aims to assess the occurrence of colonoscopy-related AEs in older individuals using the most up-to-date evidence.

## Methods

This study conducted a systematic review and meta-analysis in accordance with PRISMA (Preferred Reporting Items for Systematic Reviews and Meta-Analyses) guidelines for reporting and the AMSTAR 2 (Assessing the Methodological Quality of Systematic Reviews 2) guidelines for assessing methodological quality^[13,14]^. The protocol was recorded in the PROSPERO database.

### Search strategy

A comprehensive search was conducted on 3 June 2024, utilizing the PubMed, Embase, and Cochrane Library databases. MeSH terms and specific keywords were employed, including “older adult,” “colonoscopy,” “perforation,” “gastrointestinal bleeding,” “adverse effects,” and “mortality.” The detailed search strategy can be seen in Supplemental Digital Content, Table S1 (http://links.lww.com/JS9/D827). Additionally, a thorough review of relevant references was performed to ensure no research was overlooked. Studies were not limited by their study design or language.

### Selection criteria

The inclusion criteria were as follows: (1) population-based studies reporting one or more outcomes of colonoscopy-related AEs in older adults (≥ 65 years old); (2) colonoscopies conducted after the year 2000; and (3) studies published as full-text articles. The exclusion criteria were as follows: (1) studies investigating the safety of colonoscopy in patients with specific diseases, such as inflammatory bowel disease, hereditary polyposis, renal failure, etc; (2) studies focused on evaluating the safety of assistive techniques for colonoscopy, including AI-assisted endoscopy or endocuff; (3) studies assessing the safety of advanced colonoscopic procedures, such as double-balloon endoscopy or magnet-imaging enhanced colonoscopy; and (4) studies evaluating the safety of specific endoscopic resection techniques like ESD, EMR, EFTR, etc. Two reviewers independently scanned the titles and abstracts obtained from the electronic searches to identify potentially relevant studies and then further screened the full texts based on the inclusion criteria. Differences regarding inclusion were resolved through discussion; otherwise, a third reviewer’s decision was sought.

### Data analysis

Data extraction was conducted independently by three reviewers, focusing on key characteristics of the included studies. Any discrepancies were discussed among the reviewers, and unresolved issues were adjudicated by a senior reviewer. These characteristics encompassed details such as the first author, year and country of publication, study period, method of data collection, study type, study population, documented AEs categories, age groups, duration of follow-up, mean/median patient ages, and male/female ratios. Additionally, information regarding the total number of colonoscopies performed and patients involved was also extracted. Furthermore, considering various indications (screening and diagnosis) or purposes (therapeutic and non-therapeutic) may significantly impact the prevalence of AEs, we extracted relevant data for meta-analysis as well. In this study, “screening colonoscopy” pertained to asymptomatic individuals who underwent colonoscopy for screening purposes, whereas “diagnostic colonoscopy” pertained to symptomatic individuals undergoing colonoscopy for diagnostic reasons. “Therapeutic colonoscopy” was defined as colonoscopy with polypectomy, while “non-therapeutic colonoscopy” was referred to as colonoscopy without polypectomy. The primary outcomes assessed included colonoscopy-related perforation and bleeding, while the secondary outcomes involved mortality and other colonoscopy-related AEs (including cardiopulmonary events, cerebrovascular events, and others).

### Basic definition

In this study, colonoscopy-related AEs were defined as complications occurring within 30 days following the procedure. The term “perforation” was specifically defined as the presence of symptoms and X-ray abnormalities (such as intra-abdominal free air), necessitating hospitalization or surgical intervention. The term “bleeding” is referred to as instances of post-colonoscopy bleeding that required hospitalization, emergency department visits, repeat colonoscopy procedures, or administration of packed red blood cells. The term “colonoscopy-related mortality” was precisely defined as any death directly attributed to the colonoscopy procedure, while the term “all-cause mortality” encompassed deaths from any cause.

### Risk of bias

The Newcastle-Ottawa Scale (NOS) was utilized to assess the methodological quality of cohort and case-control studies^[15,16]^. The assessment score was evaluated based on three criteria: selection, comparability, and outcome, which were assigned ratings of 4, 2, and 3 stars, respectively. For cross-sectional studies, we employed an adapted version of the NOS developed by Herzog *et al*[17], specifically tailored for this study design. This modified assessment score also consisted of three criteria: selection, comparability, and outcome, with ratings of 5, 2, and 3 stars, respectively. Two independent reviewers conducted separate assessments to evaluate the quality of included studies, while any discrepancies in their evaluations were resolved through dialogue.

### Statistical analysis

To account for the evident heterogeneities, we employed a random-effects model proposed by DerSimonian and Laird in all analyses[18]. The prevalence of colonoscopy-related AEs was summarized using the generalized linear-mixed model (GLMM)[19], which is suitable for studies characterized by either very low or very high rates[20]. This analysis method employed binomial likelihoods for each included study, without the need to transform data or correct for zero counts. Pooled estimates with 95% confidence intervals (CIs) were presented. Statistical heterogeneity was assessed using *I*^2^ and Cochran’s Q test scores[21]. We performed subgroup analysis on prevalence rates, initially stratifying by two age groups, if data were available: 65–80 years and >80 years. This age stratification was selected due to the predominant use of 80 years old as a threshold in included studies. Risk ratios (RRs), along with their corresponding 95% CIs, were calculated to assess the differences in primary outcomes between the “65–80 years” group and the “ >80 years” group. Sensitivity analysis was performed using both the leave-one-out method and the specific group method (sample size ≥ 1000 or quality scores ≥ 6). Possible publication bias was qualitatively tested by visually inspecting the funnel plot and quantitatively examined using Egger tests when sufficient studies were identified (≥10)[22]. All statistical analyses were performed using R software (version 4.3.3, Camp Pontanezen, New Jersey, USA).

## Results

**Figure 1.**
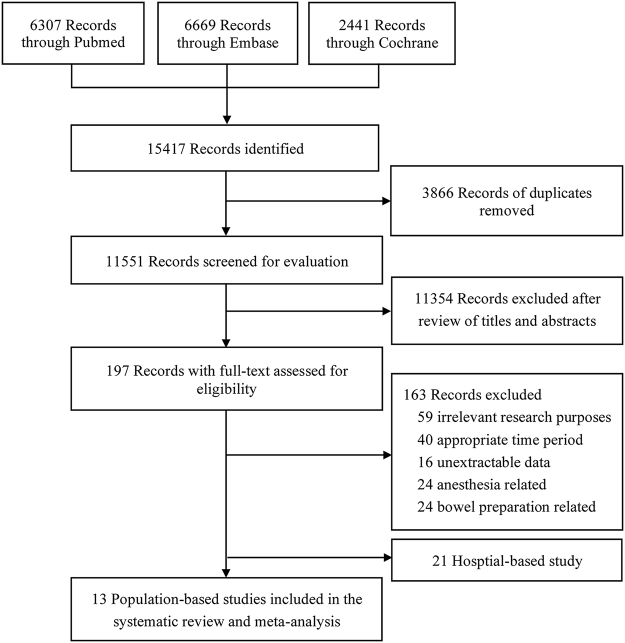
PRISMA flow diagram showing study selection process for meta-analysis.

Our initial search strategy yielded a total of 15 417 records. After excluding 3866 duplicate records and 11 354 records identified through the assessment of titles and abstracts, we reviewed the full-text of the remaining articles. Out of these, 163 articles were excluded due to various reasons such as irrelevant research purposes, inappropriate inclusion periods, focusing solely on bowel preparation-related or anesthesia-related AEs, and unextractable data. Finally, we excluded 21 hospital-based studies and included 13 population-based studies in this systematic review and meta-analysis^[6,23–34]^ (Fig. 1). Table 1 presents the characteristics of included studies. These studies encompassed a total of 829 765 colonoscopies conducted in six distinct countries. Five studies provided data for analysis in the “65–80 years” group^[23–25,30,33]^, while three studies provided data for analysis in the “ > 80 years” group^[23,30,33]^. The remaining eight studies were not grouped by age 80. The NOS and modified NOS criteria were used to evaluate all included studies. Three studies received a rating of seven stars or higher, while ten had ratings between five and six stars. Supplemental Digital Content, Tables S2 (http://links.lww.com/JS9/D828) and S3 (http://links.lww.com/JS9/D829) contain comprehensive assessment records.

**Table 1:** Characteristics of included studies

Author	Year	Country	Time period	Data collection	Study type	Study population	Adverse events documented	Age groups*	Follow-up (d)	Mean/median age (y)	Male/female ratio (*n*)	Patients (*n*)	Colonoscopies (*n*)
Halabi[23]	2023	United States	2009.01-2022.01	Retrospective	Population-based/ cross-sectional study	Outpatient	Perforation	65–80	30	78	3100/3967	7067	7067
								>80					
Yoshida[24]	2021	Japan	2005.01-2018.08	Retrospective	Population-based/ cross-sectional study	Both	Perforation, bleeding	65–80	7	NA	NA	44 021	44 021
Paszat[25]	2021	Canada	2008–2017	Prospective	Population-based/ cross-sectional study	Outpatient	Perforation, bleeding	65–80	30	NA	NA	38 751	38, 751
Kim[26]	2021	Korea	2012–2017	Retrospective	Population-based/ cohort study	Outpatient	Bleeding, CV/pulm, cerebral, morality	≥65	30	NA	40, 444/34, 448	74,892	74, 892
Causada-Calo[6]	2020	Canada	2008.04-2017.09	Retrospective	Population-based/ cohort study	Both	Perforation, bleeding, CV/pulm, morality	≥65	30	80.5	3738/3888	7626	7626
Koivogui[27]	2019	France	2008.01-2017.12	Prospective	Population-based/ cross-sectional study	Outpatient	Perforation, bleeding, CV/pulm	≥65	7	75.7	NA	575	575
Kim[28]	2019	Korea	2011.01-2011.12	Retrospective	Population-based/ case-control study	Outpatient	Perforation	≥65	30	NA	NA	3660	3660
Rim[29]	2017	Korea	2006-2013	Prospective	Population-based/ cross-sectional study	Outpatient	Perforation, bleeding, CV/pulm	≥65	30	NA	46 000/39 600	85 600	85 600
Forsberg[30]	2017	Sweden	2001–2013	Retrospective	Population-based/ cohort study	Both	Perforation, bleeding	65–80	30	NA	NA	186, 681	186,681
								>80					
Zafar[31]	2014	United States	2007.01-2008.12	Retrospective	Population-based/cohort study	Outpatient	Perforation, bleeding, CV/pulm	≥65	30	74.4	67, 586/81, 616	149, 202	149,202
Bielawska[32]	2014	United States	2000.01-2011.03	Prospective	Population-based/ cohort study	NA	Perforation	≥65	7	NA	NA	151, 210	151,210
Hamdani[33]	2013	United States	2002.01-2010.08	Retrospective	Population-based/ cross-sectional study	Both	Perforation	65–80	7	NA	NA	27,260	27, 260
								>80					
Warren[34]	2009	United States	2001.07-2005.10	Retrospective	Population-based/ cohort study	Outpatient	Perforation, bleeding, CV/pulm	≥65	30	NA	NA	53,220	53, 220

NA, not available; CV/pulm, cardiopulmonary events; cerebral, cerebrovascular events.

^*^ All groups were based on the limit of the age range of the included patients. They were divided into “65–80” group and “ > 80” group.

### Prevalence of primary outcomes

**Figure 2.**
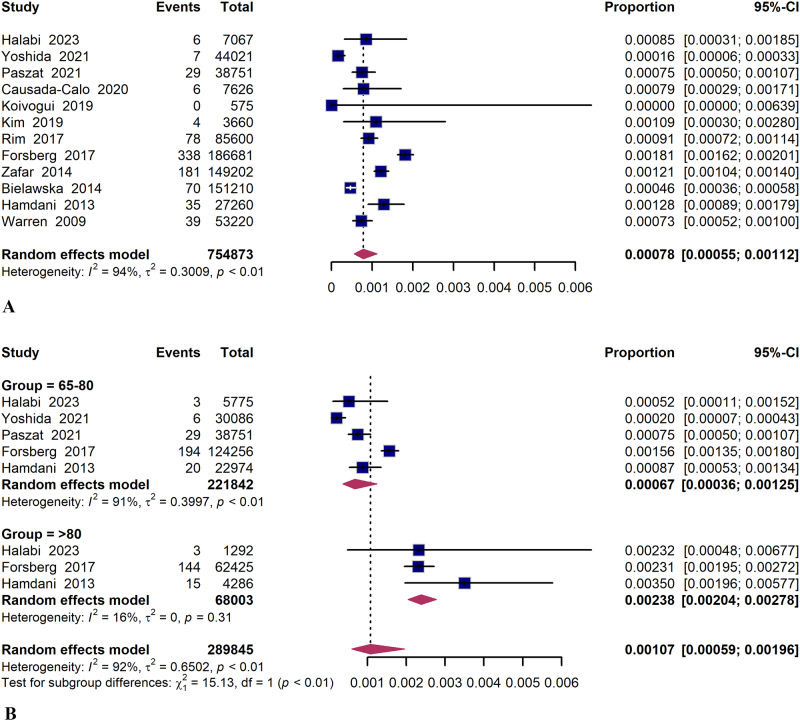
Forest plot of colonoscopy-related perforation and subgroup analysis on age stratification.

A total of 12 studies reported colonoscopy-related perforations in elderly individuals, with an overall pooled rate of 7.8 per 10 000 colonoscopies (95% CI 5.5–11.2, *I*^2^ = 94%)^[6,23–25,27–34]^ (Fig. 2A). In the “65–80 years” group, the pooled rate of perforation was estimated at 6.7 per 10 000 colonoscopies (95% CI 3.6–12.5; *I*^2^ = 91%). However, among the “ > 80 years” group, a higher pooled rate for perforation was observed at 23.8 per 10 000 colonoscopies (95% CI 20.4–27.8; *I*^2^ = 16%) (Fig. 2B). The risk of colonoscopy-related perforation was found to be significantly elevated in the “ >80 years” group compared to the “65–80 years” group (RR 2.55; 95% CI 1.15–5.66; *I*^2^ = 79%) (Supplemental Digital Content, Figure S1A, http://links.lww.com/JS9/D821).

**Figure 3.**
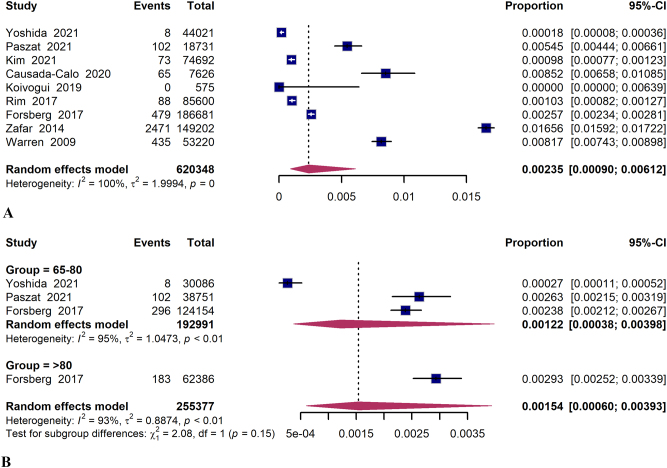
Forest plot of colonoscopy-related bleeding and subgroup analysis on age stratification.

The data on colonoscopy-related bleeding in older adults were obtained from nine studies^[6,24–27,29–31,34]^. The overall pooled rate of colonoscopy-related bleeding was 23.5 per 10 000 colonoscopies (95% CI 9.0–61.3, *I*^2^ = 100%). In the “ >80 years” group, the pooled rate of bleeding was higher at 29.3 per 10 000 colonoscopies (95% CI 25.2–33.9; *I*^2^ = 0%), compared to the “65–80 years” group with a rate of 12.2 per 10 000 colonoscopies (95% CI 3.8–39.8; *I*^2^ = 95%) (Fig. 3). The risk of colonoscopy-related bleeding was found to be significantly elevated in the “ > 80 years” population compared to the “65–80 years” population (RR 1.23; 95% CI 1.02–1.48; *I*^2^ = 0%) (Supplemental Digital Content, Figure S1B, http://links.lww.com/JS9/D821). The summary of pooled results can be seen in Table 2.

**Table 2:** Summary of pooled results

	Perforation		Bleeding	
	Effect size * (95% CI)	*I*^2^, %	Effect size * (95% CI)	*I*^2^, %
Overall	0.078 (0.055–0.112)	94	0.235 (0.090–0.613)	100
Age of individuals				
65–80 years old	0.067 (0.036–0.125)	91	0.122 (0.038–0.398)	95
>80 years old	0.238 (0.204–0.278)	16	0.293 (0.252–0.339)	0
>80 vs. 65–80 years old	2.55 (1.15–5.66)	79	1.23 (1.02–1.48)	0
Purpose of colonoscopy				
Non-therapeutic colonoscopy	0.004 (0.001–0.012)	0	0.005 (0.000–1.747)	0
Therapeutic colonoscopy	0.015 (0.003–0.079)	79	0.071 (0.049–0.103)	0
Indication for colonoscopy				
Screening colonoscopy	0.085 (0.071–0.102)	0	0.270 (0.090–0.810)	99
Diagnostic colonoscopy	0.180 (0.162–0.200)	1	0.160 (0.081–0.313)	98

CI, confidence interval.

^*^ The risk ratio (RR) is used as the effect size for comparison between the “65–80 years old” group and the “ > 80 years old” group; effect sizes in all other blanks are prevalence rates (%).

### Prevalence of secondary outcomes

In addition to primary outcomes, a total of six included studies reported other AEs (Supplemental Digital Content, Table S4, http://links.lww.com/JS9/D830). Due to significant variations in inclusion criteria and differing definitions employed across the studies, we utilized descriptive approaches to characterize these AEs. To summarize, we categorized these events into four groups: cardiopulmonary events, cerebrovascular events, all-cause mortality, and other events (nausea and vomiting).

Among these six studies, a total of 6364 cardiopulmonary events among 286 471 colonoscopies were observed^[6,26,27,29,31,34]^. Waren *et al*[34] and Zafar *et al*[31] reported cardiovascular events within the same category. The main events documented included 1353 cases of myocardial infarction/angina, 3297 cases of arrhythmias, 2179 cases of heart failure, 659 cases of cardiac/respiratory arrest, and 1141 cases of syncope/hypotension/shock out of a total of 202 422 colonoscopies performed. The remaining four studies only documented the overall prevalence of cardiopulmonary events without differentiating among specific categories^[6,25,27,29]^.

No studies have reported colonoscopy-related mortality data. Three studies provided information on all-cause mortality, documenting a total of 534 deaths attributed to various causes out of 231 720 colonoscopies performed^[6,26,31]^. Only one study reported the occurrence of cerebrovascular events, with 31 cases of cerebral hemorrhage /infarction observed among the 74 892 colonoscopies performed[26]. Additionally, incidences of nausea and vomiting were reported by Waren *et al*[34] and Zafar *et al*[31], with a total number of 2082 cases identified out of the overall cohort comprising 202 422 colonoscopies.

### Subgroup analysis

The occurrence rates of perforation (0.015% vs. 0.004%) and bleeding (0.071% vs. 0.005%) were significantly higher in therapeutic colonoscopies compared to non-therapeutic colonoscopies, as indicated by subgroup analysis based on the purpose of colonoscopies (Supplemental Digital Content, Figures S2 http://links.lww.com/JS9/D822 and S3 http://links.lww.com/JS9/D823). Alternatively, subgroup analysis based on the indication of colonoscopies revealed that screening colonoscopies had estimated overall rates of perforation and bleeding at 0.085% and 0.270%, respectively, while diagnostic colonoscopies showed pooled rates of perforation and bleeding at 0.180% and 0.160%, respectively (Supplemental Digital Content, Figures S4 http://links.lww.com/JS9/D824 and S5 http://links.lww.com/JS9/D825).

### Sensitive analysis and publication bias

We conducted a sensitivity analysis by systematically removing one study at a time to assess the influence of each individual study on the findings. The results indicated that none of the studies had a significant impact on the overall effect of all primary outcomes. Additionally, we performed additional analyses to evaluate the potential effects of sample size and study quality, revealing consistent results (Supplemental Digital Content, Table S5, http://links.lww.com/JS9/D831). No substantial asymmetry was observed in the funnel plots for both perforation and bleeding (Supplemental Digital Content, Figure S6, http://links.lww.com/JS9/D826). Moreover, statistical tests using Egger’s regression did not yield any statistically significant findings (*P* = 0.17 for perforation and *P* = 0.71 for bleeding).

## Discussion

We conducted a comprehensive evidence-based study to determine the prevalence of AEs related to colonoscopy in the elderly population over the past two decades. The existing literature on this topic was limited, with only one meta-analysis focusing on colonoscopies performed in the late 1990s[35]. Given the statistical limitations and restricted data sources in that study, it is essential to update and expand their findings. Furthermore, considering the significant increase in demand for colonoscopies among older individuals since 2000, analyzing data from the previous two decades becomes imperative. Our current study provides a broader range of information by specifically stratifying age groups for older individuals and examining different purposes or indications for colonoscopy that have not been previously discussed.

In our study, the pooled rate of perforation (0.078%) observed was higher than that reported by ASGE for the general population (0.058%)[4]. By employing the same statistical methodology utilized in the ASGE study, we would have achieved a higher pooled perforation rate of 0.083%. The elevated perforation rate can be attributed to several factors. First, the increased incidence of colonic wall fragility associated with aging renders it more susceptible to perforation during procedures involving stretching, manipulation, or resection of the bowel[35]. Second, a higher prevalence of comorbid conditions such as diverticulosis and previous abdominal surgeries in older adults can also increase procedural technical difficulty^[36,37]^. As summarized in Supplemental Digital Content, Table S6 (http://links.lww.com/JS9/D832), although the pooled perforation rates were slightly higher in the elderly populations than in general populations, the overall incidence remained low.

Interestingly, our study revealed a slightly lower bleeding rate (0.235%) compared to the reported prevalence in the general population (0.246%)[4]. This discrepancy may be attributed to variations in data transformation methods employed during meta-analysis[20]; when employing the same methodology as utilized in the ASGE study, we found that the pooled bleeding rate among elderly individuals was 0.340%. The aging process is associated with a decline in the gastrointestinal mucosal integrity and tissue regenerative capacity, thereby increasing the vulnerability of colonic mucosa to injury during endoscopic procedures such as polypectomy or biopsy^[38,39]^. Furthermore, despite meticulous management regarding procedural timing, the frequent use of anticoagulant and antiplatelet medications may further increase susceptibility to bleeding during invasive colonoscopic procedures[40]. Again, as summarized in Supplemental Digital Content, Table S6 (http://links.lww.com/JS9/D832), the overall incidence of bleeding is low in the elderly populations.

Importantly, compared to the study conducted by Day *et al*, there has been a notable decrease in both the prevalence of bleeding and perforation associated with colonoscopy in elderly individuals over the past two decades[35]. This improvement can be attributed primarily to advancements in colonoscopic technology. Modern colonoscopes offer enhanced flexibility and superior imaging capabilities and enable more precise interventions^[39,41]^. Furthermore, there has been an increased emphasis on employing less invasive techniques for polyp removal, such as cold snare polypectomy, which is associated with a reduced risk of bleeding compared to traditional hot snare methods^[42,43]^. The widespread adoption of prophylactic measures like endoscopic clips for preventing post-polypectomy bleeding and perforation has also contributed to these positive outcomes. Last, there is now a better understanding of the risks associated with colonoscopy, leading to more careful selection of candidates. Clinicians are now inclined toward optimizing medical management before performing colonoscopy by adjusting anticoagulant therapy and managing comorbid conditions that could increase the risk of complications[44].

Our meta-analysis reveals a significant disparity in AE rates between non-therapeutic and therapeutic colonoscopies, as well as screening and diagnostic colonoscopies. While therapeutic colonoscopies inherently carry a higher risk of colonic wall damage, leading to complications such as bleeding and perforation, the overall risks remain low and manageable. In elderly patients, these risks can be influenced by age-related changes in tissue integrity and existing comorbidities, but careful pre-procedural planning can help minimize complications. A comprehensive evaluation of the patient’s overall health, comorbidities, and medication usage is essential for optimizing procedural safety[45]. For instance, long-term medications, such as anticoagulants and antiplatelet agents, could increase the risk of bleeding, while conditions like cardiovascular disease or diabetes might enhance susceptibility to procedural stress or hinder recovery. Although the studies included in our meta-analysis did not provide data on these variables, it highlights an important area for further research. Such investigations would contribute to developing better risk stratification tools and more personalized protocols for elderly patients, ultimately improving the safety and efficacy of therapeutic colonoscopies. Diagnostic colonoscopies, compared to screening procedures, often involve more extensive tissue sampling or removal of larger lesions, which may contribute to a slightly higher risk of perforation^[46,47]^. Additionally, the increased incidence of bleeding during screening colonoscopies could be attributed to a heightened probability of detecting and removing polyps during these procedures. Polypectomy, particularly when multiple or large polyps are removed, increases the risk of post-procedural bleeding[48]. Clinicians should be mindful of potential complications in elderly patients, particularly regarding bleeding risk when polypectomy is required. Advanced endoscopic techniques may also minimize the risks of perforation and bleeding during both diagnostic and screening colonoscopies on the basis of a clearer investigation[4].

Due to possible variations in the description and classification of cardiopulmonary AEs across studies, we refrained from pooling the data. However, our review indicates a relatively high prevalence of cardiopulmonary AEs related to colonoscopy. Previous evidence has demonstrated a direct correlation between age and cardiovascular events among older individuals, with those over 75 years experiencing a four-fold increase compared to younger individuals^[6,34]^. Elderly patients are more likely to have pre-existing cardiopulmonary conditions that heighten their vulnerability to cardiopulmonary stress during colonoscopy induced by sedation, changes in intravascular volume, and physiological response to bowel preparation. Physical stress imposed by colonoscopy itself may also exacerbate existing cardiopulmonary conditions. Given the increased risk of cardiopulmonary events, clinicians should conduct thorough pre-procedural assessments to identify higher-risk patients[49]. During the procedure, careful monitoring of vital signs is essential for maintaining stable hemodynamics. Sedative agents and dosing should be tailored to minimize cardiovascular stress[49]. Post-procedural monitoring should be extended in elderly patients who experience significant hemodynamic changes during the procedure.

Although our study found an increased risk of perforation and bleeding in elderly patients undergoing colonoscopy, these risks remain relatively low compared to other endoscopic procedures such as ESD and EMR[4]. In other words, while colonoscopy in older adults does carry some risks, it still maintains a relatively favorable safety profile, particularly when appropriate preventive measures are taken. To further enhance safety, comprehensive pre-procedure evaluations, customized sedation protocols, and careful monitoring during and after the procedure are essential. Informed consent discussions should clearly outline the specific risks and benefits of colonoscopy, empowering patients to make well-informed decisions about their care. For some elderly patients, particularly those with significant comorbidities or frailty, alternative screening methods such as fecal immunochemical tests (FIT) and CT colonography may offer safer options^[50,51]^.

Our study has some unique features. First, the data included in our study primarily originated from high-resource regions, such as North America, Europe, and Asian countries, which often feature advanced healthcare systems and standardized procedural practices. Consequently, the AE rates reported in our study may reflect the characteristics of regions with safer colonoscopic procedures. Future studies should investigate colonoscopy-related AEs across diverse regions to better represent the global safety profile in elderly populations. Second, our study included population-based studies to enhance the external validity of the findings by representing real-world settings. In our view, population-based studies better reflected real-world data by encompassing a broader range of patients, including those from diverse regions and healthcare conditions, thereby minimizing the selection bias that may arise from single-hospital or medical-center studies. These studies usually rely on national or regional databases with standardized data collection.

Our study has several limitations. First, due to the limited number of included studies and the complexity in interpreting results, we refrain from presenting analysis findings for alternative age thresholds (such as 70, 75, or 90 years old). Further large-scale research and national data are necessary to supplement the analysis using these cut-off values. Second, although population-based studies offer advantages such as larger sample sizes that reduce bias risk, they often lack detailed information on intraoperative anesthesia details, endoscopist proficiency levels, and endoscopic instruments used. Finally, insufficient data availability from the included studies prevented us from conducting subgroup analyses on certain individual parameters such as health condition, comorbidities, and long-term medication factors which typically have potential implications for AEs associated with colonoscopy in elderly patients.

## Conclusions

We conducted a comprehensive analysis on the prevalence of AEs related to colonoscopy in older adults. Overall, the AE rates remain low. However, we emphasize the importance of enhancing safety protocols to further minimize risks, ensuring that the benefits of colonoscopy continue to outweigh the risks, especially for patients over the age of 80.

## Data Availability

All data are provided in the article and in the appendix.

## Research registration unique identifying number (UIN)

PROSPERO ID: CRD42024520183.
